# Graph‐Based Pangenome of *Actinidia chinensis* Reveals Structural Variations Mediating Fruit Degreening

**DOI:** 10.1002/advs.202400322

**Published:** 2024-05-17

**Authors:** Yingzhen Wang, Pengwei Li, Yanyan Zhu, Feng Zhang, Sijia Zhang, Yan He, Ying Wu, Yunzhi Lin, Hongtao Wang, Wangmei Ren, Lihuan Wang, Ying Yang, Runze Wang, Pengpeng Zheng, Yongsheng Liu, Songhu Wang, Junyang Yue

**Affiliations:** ^1^ Anhui Province Key Laboratory of Horticultural Crop Quality Biology School of Horticulture Anhui Agricultural University Hefei 230036 China; ^2^ Ministry of Education Key Laboratory for Bio‐resource and Eco‐environment College of Life Science State Key Laboratory of Hydraulics and Mountain River Engineering Sichuan University Chengdu 610064 China; ^3^ School of Forestry Science and Technology Lishui Vocational and Technical College Lishui 323000 China

**Keywords:** Actinidia chinensis, fruit degreening, haplotype‐resolved, pangenome, structural variations

## Abstract

Fruit ripening is associated with the degreening process (loss of chlorophyll) that occurs in most fruit species. Kiwifruit is one of the special species whose fruits may maintain green flesh by accumulating a large amount of chlorophyll even after ripening. However, little is known about the genetic variations related to the fruit degreening process. Here, a graph‐based kiwifruit pangenome by analyzing 14 chromosome‐scale haplotype‐resolved genome assemblies from seven representative cultivars or lines in *Actinidia chinensis* is built. A total of 49,770 non‐redundant gene families are identified, with core genes constituting 46.6%, and dispensable genes constituting 53.4%. A total of 84,591 non‐redundant structural variations (SVs) are identified. The pangenome graph integrating both reference genome sequences and variant information facilitates the identification of SVs related to fruit color. The SV in the promoter of the *AcBCM* gene determines its high expression in the late developmental stage of fruits, which causes chlorophyll accumulation in the green‐flesh fruits by post‐translationally regulating AcSGR2, a key enzyme of chlorophyll catabolism. Taken together, a high‐quality pangenome is constructed, unraveled numerous genetic variations, and identified a novel SV mediating fruit coloration and fruit quality, providing valuable information for further investigating genome evolution and domestication, QTL genes function, and genomics‐assisted breeding.

## Graphical abstract

This study builds a graph‐based pangenome of kiwifruit (*Actinidia chinensis*) by assembling 14 chromosome‐scale haplotype‐resolved genome from seven representative cultivars. The pangenome facilitates the identification of a structure variation (SV) in the promoter of the *AcBCM* gene. The SV is correlated with the green‐flesh fruit in *Actinidia chinensis*.

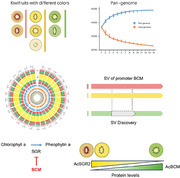

## 1 Introduction

Fruit ripening is the final stage of fruit development that is assocated with the multiple alterations of fruit color, taste, aroma, and firmness.^[^ ^1^ ^]^ In most species, fruits display a degreening phenomenon (loss of chlorophyll) during the ripening process. Chlorophyll has been proven to be an important nutrient element.^[^ ^2^ ^]^ Unfortunately, the chlorophylls synthesized in diet fruits are degraded during the ripening process. Kiwifruit (*Actinidia* L.) is one of special fruits that can maintain green flesh (high contents of chlorophyll) even after ripening.^[^ ^3^ ^]^ The degreening process is suppressed in some species or cultivars of kiwifruit. However, the underlying mechanisms remain largely unknown.

Kiwifruit belonging to woody perennial species is a globally popular fruit with particularly high vitamin C content. Although the genus *Actinidia* has 54 species, only four species (*A. chinensis*, *A. deliciosa*, *A. eriantha*, and *A. arguta*) have already been bred into commercial cultivars.^[^ ^4^ ^]^ Fruit color is an important agronomic trait that affects the popularity and commercial value. Among the four commercialized species, *A. chinensis* displays the richest diversity of fruit colors including green, yellow, red, and the combinations.^[^ ^5^ ^]^ Generally, three main pigments including chlorophyll (green), carotenoid (yellow), and flavonoids (red) contribute to fresh coloration in kiwifruit.^[^ ^3^, ^5^, ^6^ ^]^ Although the previous studies revealed many genes involved in regulating the accumulation of these pigments, little is known about the genetic variations, such as structural variations (SVs), that mediate fruit coloration in kiwifruit. The identification and application of SVs are impeded by the lack of pangenome in kiwifruit.

The progress of high‐throughput sequencing technologies has greatly accelerated the identification of candidate genetic variations including small insertions/deletions (InDels) and single nucleotide polymorphisms (SNPs).^[^ ^7^, ^8^ ^]^ Recently, a number of studies based on comparative and functional genomics of plants have suggested that the larger structural variations (SVs) throughout the genomes, another important source of genetic variations, genetically control agronomic traits and play pivotal roles in genome evolution.^[^ ^9^ ^]^ Although the longer reads generated via the cutting‐edge third‐generation sequencing platforms allow detecting SVs with higher sensitivity and more accuracy than short reads, there are still major limitations in capturing the whole picture of genome‐wide genetic diversity by using the alignments against one or a few reference genomes due to the widespread presence/absence variants (PAVs).^[^ ^10^ ^]^ Additionally, it's a big challenge to reveal the genotypes of different alleles at individual loci with conventional linear reference genome.^[^ ^11^ ^]^ Therefore, it is indispensable to construct a graph‐based pangenome of a species or even a larger taxon depending on the development of multiple representative reference genomes. The pan genome was first established in bacterial research, referring to a single individual containing only a portion of the genes of its species.^[^ ^12^ ^]^ Recently, an increasing number of pangenome studies have been conducted on horticultural crops, such as tomato,^[^ ^13^, ^14^, ^15^ ^]^ *Brassica napus*,^[^ ^16^ ^]^ strawberry,^[^ ^17^ ^]^ cucumber,^[^ ^18^ ^]^ citrus,^[^ ^19^ ^]^ apple,^[^ ^20^ ^]^ tea plant,^[^ ^21^ ^]^ watermelon,^[^ ^22^ ^]^ which have uncovered tremendous genomic divergence/variation at a population scale and shed new light on the genetic basis of evolution, domestication, and improvement.

Based on the reference genome of *A. chinensis* cv. “Hongyang” (2n = 2x = 58),^[^ ^4^, ^23^, ^24^, ^25^ ^]^ several research groups using Illumina short reads have performed the systematic investigation of genomic sequence variations such as InDels and SNPs, and further revealed their association with gender identification,^[^ ^26^ ^]^ fruit number and weight.^[^ ^27^ ^]^ In addition, natural diversity of SNPs across 25 taxa suggested the evolutionary history and reticulate speciation of kiwifruit.^[^ ^28^ ^]^ Recently, two sex‐determinant genes *SyGl* ^[^ ^9^ ^]^ and *FrBy* ^[^ ^29^ ^]^ were identified to be located in the Y‐specific sequences that are only present in male genomes^[^ ^29^, ^30^ ^]^ but absent in female genomes,^[^ ^4^, ^31^ ^]^ indicating the pivotal role of genetic variations in determining agronomic traits. However, the low quality and small number of genomes hampered a complete characterization of genetic variations among different kiwifruit accessions, especially SVs, emphasizing the necessity of developing additional high‐quality genome assemblies from more kiwifruit accessions/varieties.^[^ ^31^, ^32^, ^33^ ^]^

In this study, we have *de novo* assembled the high‐quality chromosome‐scale and haplotype‐resolved genomes of six representative *A. chinensis* varieties or lines and obtained a total of 12 haplotypes. Together with two haplotypes from the T2T reference genome of *A. chinensis* cv. “Hongyang”,^[^ ^31^ ^]^ we construct a graph‐based pangenome of *A. chinensis* and disclose millions of genetic variations that are hard to be detected by aligning with a single reference genome. Our pangenome facilitates the identification of large SVs potentially related to fruit coloration including green flesh formation. Based on the constructed pangenome graph, we characterized an SV exclusively present in the promoter of *A. chinensis BALANCE of CHLOROPHYLL METABOLISM* (*AcBCM*)^[^ ^34^ ^]^ in green‐fleshed cultivars. The SV leads to the high expression of *AcBCM*, which causes chlorophyll accumulation via inhibiting its decomposition during the ripening process.

## 2 Results

### 2.1 The *De Novo* Assembly of Chromosome‐Scale and Haplotype‐Resolved Genomes of Six Kiwifruit Varieties

**Figure 1:**
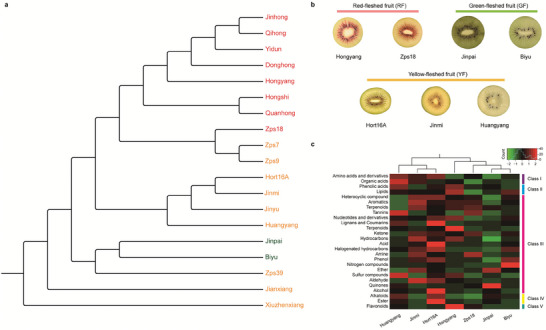
The seven representative varieties selected from a total of 19 domesticated kiwifruits in *A. chinensis*. a) Phylogenetic tree was constructed based on genome‐wide SNPs using *A. eriantha* ‘White’ as an outgroup. b) Three distinct groups categorized by fruit colors of the seven kiwifruit varieties. c) Clustered heatmap of differential metabolites found by metabolomics analysis.

Based on the phylogenetic relationship, we selected seven representative varieties from a total of 19 domesticated lines derived from *A. chinensis*, most of which are commercially cultivated varieties (**Figure** **1**a). Briefly, they could be categorized into three distinct groups: red‐fleshed fruit (RF) group with two lines “Hongyang” and “Zps18”, yellow‐fleshed fruit (YF) group with three lines “Hort16A”, “Huangyang” and “Jinmi”, and green‐fleshed fruit (GF) group with two lines “Biyu” and “Jinpai” (Figure 1b). Furthermore, our grouping information was consistent with the hierarchical clusters based on secondary metabolism detected in their ripe fruits, implying that the biosynthesis and accumulation of metabolites are highly determined by the genetic diversity of these varieties (Figure 1c).

In addition to the haplotype‐resolved genome assembly of “Hongyang” available,^[^ ^31^ ^]^ we further performed PacBio HiFi sequencing for the other six varieties and produced 25.3–34.0 Gb of long reads, representing 40.9–54.9× genome coverages (Table S1, Supporting Information). The contigs were individually *de novo* assembled by hifiasm (https://github.com/chhylp123/hifiasm) using a haplotype phasing strategy. A total of 174–777 contigs were generated, with N50 values ranging from 15.6 to 20.4 Mb (**Table** **1**). Compared to the three previously existing kiwifruit genomes,^[^ ^4^, ^24^, ^57^ ^]^ a noticeably higher N50 value coupled with a relatively fewer number of contigs was obtained. Almost the same size between chromosome average length and contigs N50 value indicated that the haplotype‐resolved assemblies are of high quality. The contigs with a larger size (>= 100 Kb) were then clustered, ordered, connected, and orientated into 29 pseudo‐chromosomes based on their alignments against the T2T reference genome Hongyang v4.0^[^ ^31^ ^]^ by using the quarTeT software (http://www.atcgn.com:8080/quarTeT/home.html). The chromosome identifiers were also assigned and sorted by referring to Hongyang v4.0.

**Table 1:** Summary statistics of kiwifruit genome assemblies.

Assembly	Variety	Species	Group	Genome size [Mb]	No. of contigs	Contig N50 [Mb]	Genome complete^a^ ^)^ [%]	No. of genes	Gene complete^b^ ^)^ [%]	TE content [%]	LAI value
Biyu.hap1	Biyu	*A. chinensis*	GF	602.0	777	19.6	99.4	46,073	96.5	42.5	15.88
Biyu.hap2	Biyu	*A. chinensis*	GF	597.9	337	18.8	98.8	46,634	96.2	41.5	15.97
Hongyang.hap1^c^ ^)^	Hongyang	*A. chinensis*	RF	606.1	267	19.0	99.3	45,809	96.9	42.3	16.38
Hongyang.hap2^c^ ^)^	Hongyang	*A. chinensis*	RF	599.6	208	18.0	99.3	45,434	96.8	41.3	15.98
Hort16A.hap1	Hort16A	*A. chinensis*	YF	602.7	711	19.2	99.4	44,336	97.2	42.2	16.27
Hort16A.hap2	Hort16A	*A. chinensis*	YF	601.3	351	19.3	99.3	46,230	96.7	42.0	16.48
Huangyang.hap1	Huangyang	*A. chinensis*	YF	604.7	574	18.9	99.1	45,982	96.5	41.8	15.99
Huangyang.hap2	Huangyang	*A. chinensis*	YF	601.0	255	17.6	99.3	45,004	96.8	42.0	16.18
Jinmi.hap1	Jinmi	*A. chinensis*	YF	604.5	364	20.4	99.5	45,473	96.7	41.9	15.74
Jinmi.hap2	Jinmi	*A. chinensis*	YF	595.6	174	18.6	99.0	45,101	95.7	41.7	16.24
Jinpai.hap1	Jinpai	*A. chinensis*	GF	603.3	390	18.1	99.4	46,244	96.7	41.9	16.29
Jinpai.hap2	Jinpai	*A. chinensis*	GF	592.7	264	15.6	99.1	45,675	96.4	41.4	15.93
Zps18.hap1	Zps18	*A. chinensis*	RF	602.7	532	18.1	99.3	45,873	95.7	41.8	16.81
Zps18.hap2	Zps18	*A. chinensis*	RF	603.1	253	20.1	99.4	45,481	96.5	41.9	15.95

^a)^ Based on BUSCO assessment of the whole genome;

^b)^ Based on BUSCO assessment of the protein‐coding genes;

^c)^ Data from the published T2T and gap‐free genome of Hongyang v4.0 (Yue et al., 2023).

So far, we have acquired 14 high‐quality chromosome‐scale genome assemblies with total sizes ranging from 592.7 to 606.1 Mb (Table 1). The genome BUSCO scores^[^ ^43^ ^]^ were all above 98%, and the LTR assembly index (LAI) values^[^ ^44^ ^]^ were all above 15, indicating both high completeness and continuity in these assemblies (Table 1; Table S2, Supporting Information). A total of 41.3–42.5% repeat sequences were identified, with long terminal repeat retrotransposons (LTR‐RTs) being the richest, accounting for 21.8% to 23.6%. (Table 1; Data S1, Supporting Information). We also identified 326728–332523 genome‐wide simple sequence repeats (SSRs) and classified them into seven types (Table S3, Supporting Information). In these assemblies, a total of 44336 to 46634 genes were identified, with gene lengths ranging from 5234 to 5,522 bp and CDS lengths from 1161 to 1,199 bp (Table S4, Supporting Information). The gene BUSCO scores ranging from 95.7% to 97.2% (Table 1; Table S5, Supporting Information), indicated that the results of gene structure annotation were high quality and suitable for downstream analyses.

### 2.2 Protein‐Coding Gene‐Based Pangenome of the Kiwifruit

**Figure 2:**
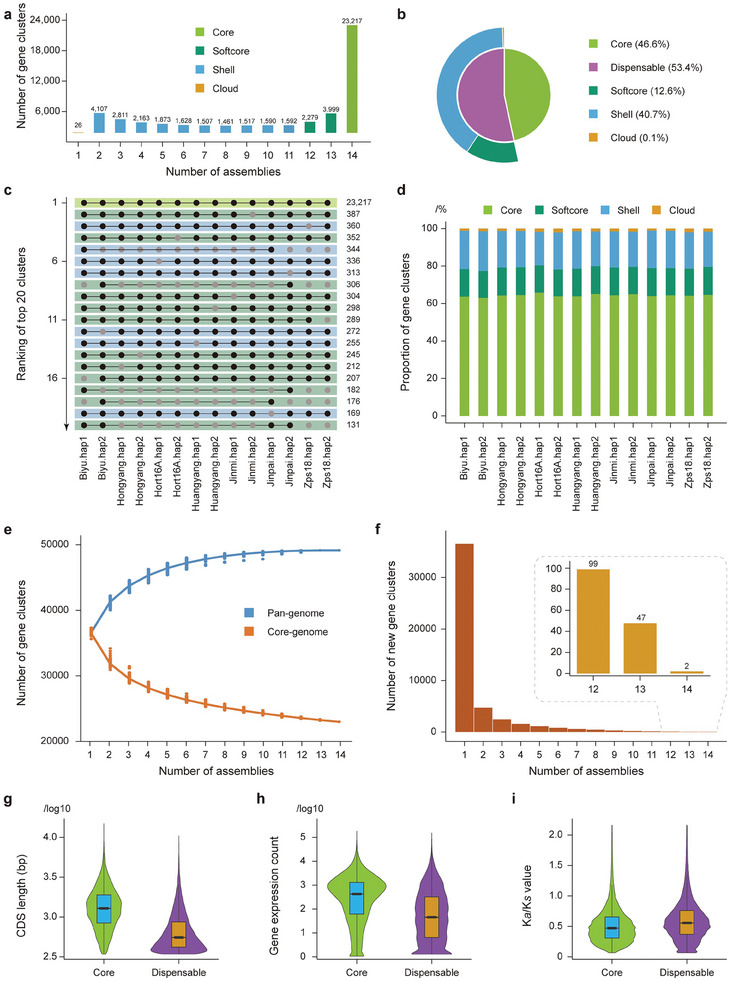
Composition of kiwifruit pan‐genome. a) Number of gene families represented versus number of genome assemblies. b) Composition of core, softcore, shell and cloud genes in the kiwifruit pan‐genome. c) The top 20 pan‐genome clusters ranked by membership of genes. d) Composition of core, softcore, shell and cloud genes per genome assembly. e) Statistics of the pan and core gene clusters. f) Statistics of the new pan gene clusters along with new genome assemblies added. g) Violin plots showing CDS lengths of core and dispensable genes. h) Violin plots showing gene expression levels of core and dispensable genes. i) Violin plots showing *Ka*/*Ks* values of core and dispensable genes.

Using OrthoFinder (https://github.com/davidemms/OrthoFinder), we clustered 639349 genes from the 14 assemblies into 49770 non‐redundant pan‐gene clusters. Based on the distribution of these gene clusters in all assemblies, a total of 23217 genes (46.6%) core (genes present in all 14 assemblies) were categorized as core gene and 26553 (53.4%) genes were categorized as dispensable ones (genes absent in at least one assembly) (Table S6 and Data S2, Supporting Information). These dispensable clusters were further categorized into 6278 (12.6%) softcore (genes in those clusters present in more than 80% assemblies), 26 (0.1%) cloud (genes in those clusters present in only one assembly), and 20249 (40.7%) shell (genes in the rest clusters) ones based on their presence in each assembly (**Figure** **2**a,b). The top 20 pangenome clusters ranked by the membership of genes from combinations of different assemblies were displayed, among which five were from shell clusters, 14 were from softcore clusters, and one was the core cluster (Figure 2c; Data S3, Supporting Information). On average, each individual kiwifruit genome assembly consisted of 64.3% core genes, 14.7% softcore genes, 19.5% shell genes, and 1.4% cloud genes (Figure 2d). The results of simulating the pangenome size indicate that when the number of assemblies exceeds eleven, the total gene number and core gene number in the pangenome approach stability, suggesting that the constructed pangenome has reached saturation (Figure 2e). Besides, only two new gene clusters were obtained when adding the 14th assembly (Figure 2f), indicating the representation of the seven kiwifruit varieties sampled in our study.

The length and expression levels of core genes were significantly higher than dispensable genes (Figure 2 g,h; Table S7, Supporting Information). The non‐synonymous/synonymous substitution ratio (*K_a_*/*K_s_)* of dispensable genes was higher than core genes, indicating that the dispensable genes may be subject to strong selective pressure (Figure 2i; Table S7, Supporting Information). The result of gene ontology (GO) enrichment indicated that core genes are primarily involved in the basic biological processes such as the regulation of cell differentiation, transcription regulatory region, transcription factor activity, and sequence‐specific DNA binding (Table S8, Supporting Information). The dispensable genes were involved in primary and secondary metabolism such as the sucrose metabolic process, quercetin 3‐O‐glucosyltransferase activity, and anthocyanidin 3‐O‐glucosyltransferase activity (Table S9, Supporting Information). Meanwhile, the KEGG analysis showed that core genes are found to be predominantly enriched in pathways such as other glycan degradation, amino sugar and nucleotide sugar metabolism, and zeatin biosynthesis (Table S10, Supporting Information), whereas dispensable genes exhibit significant enrichments in those pathways of steroid hormone biosynthesis, nitrogen metabolism and isoflavonoid biosynthesis (Table S11, Supporting Information). All these findings suggest that core genes are comparatively conserved, whereas dispensable genes display higher evolution rates and may acquire new functions to adapt to various environmental conditions or selection pressures.

### 2.3 Structural Variation Feature of Kiwifruit Genomes

**Figure 3:**
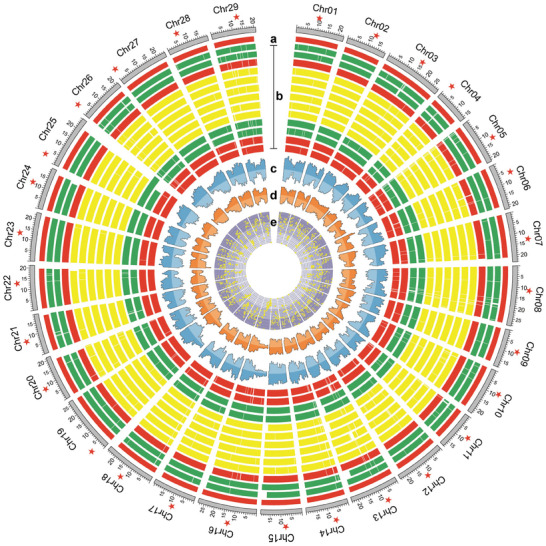
Collinear alignment blocks, genetic variants and graph‐based pan‐genome of kiwifruit. a) Pseudo‐chromosomes of the kiwifruit reference genome HY4P (Hongyang.hap1) with their centromeres marked by red pentagrams. b) Intergenomic collinear blocks of the rest genome assemblies against the HY4P reference, where non‐synteny regions are shown by white bars. From upper to lower, the assemblies are Biyu.hap1, Biyu.hap2, Hongyang.hap2, Hort16A.hap1, Hort16A.hap2, Huangyang.hap1, Huangyang.hap2, Jinmi.hap1, Jinmi.hap2, Jinpai.hap1, Jinpai.hap2, Zps18.hap1, and Zps18.hap2. c) Density distribution of large InDels (Insertion outside and Deletion inside) from the pan‐genome. d) Density distribution of small InDels (Insertion outside and Deletion inside) from the pan‐genome. e) Density distribution of SNPs from the pan‐genome.

**Figure 4:**
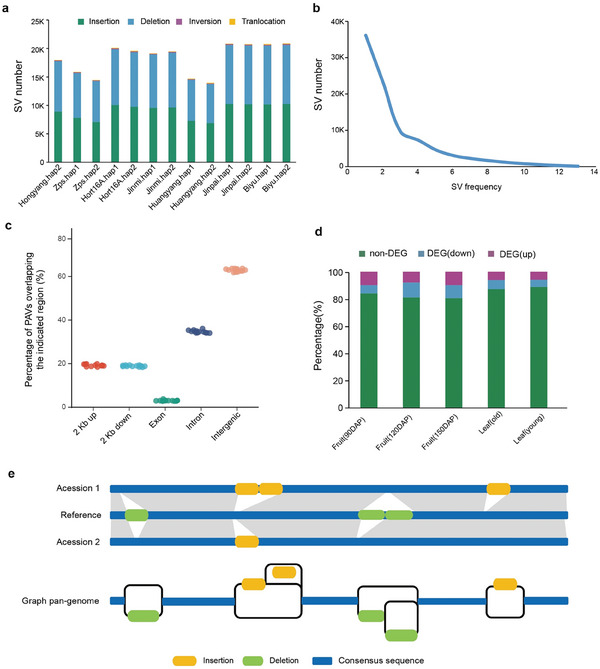
Genetic Variations from seven kiwifruit Genomes. a) The number of SVs in each kiwifruit accession. b) The frequency of SVs in kiwifruit accessions. c) Percentages of SVs sharing overlap with different genomic regions in the seven kiwifruit accessions. Each point is one accession. d) Percentage of the genes containing SV in the promoter regions that were downregulated, upregulated and not differentially expressed between RF cultivar ‘Hongyang’ and GF ‘Biyu’. e) Kiwifruit graph‐based pan‐genome that integrated sequences and positions of SVs while preserves the linear reference coordinates.

Through global alignments of intergenomic collinear blocks (**Figure** **3**a,b), a thorough inspection of genetic variations including structural variations (SVs) (in terms of large InDels that are ≥50 bp in size, inversions, and translocations) (Figure 3c), small insertions and deletions (InDels that are <50 bp in size) (Figure 3d) and single nucleotide polymorphisms (SNPs) (Figure 3e) were detected in seven kiwifruit accessions. A total of 13914 to 20883 SVs were identified, and their sizes accounted for 1.98% to 4.28% of each genome (**Figure** **4**a). Among the four types of SVs, the numbers of presence and absence were nearly equal, and they were significantly greater than the numbers of inversions or translocations (Figure 4a). In the seven kiwifruit accessions, the number of SVs in “Zps18” or “Huangyang” was significantly lower than in other accessions, while the green‐fruits group had the largest number of SVs (Figure 4a), indicating their differential relationship with “Hongyang”. We analyzed the distribution frequency of SVs in seven kiwifruit accessions and found that the majority of SVs are concentrated within individual lines (Figure 4b), suggesting that many unique SVs are present across different accessions. In addition, we examined the genomic locations of structural variations (SVs) and found that the majority of SVs are located in non‐coding regions, particularly in intergenic (>60%) and intronic (>34%) regions, with only a small proportion found in exonic (<4%) regions (Figure 4c). By integrating the transcriptomic data from RF cultivar “Hongyang” and GF “Biyu”, we found that ≈11%–19% of genes with SVs in their promoters exhibit significant changes in gene expression levels between the two cultivars (Figure 4d).

We merged the SVs (including presence and absence) from the seven kiwifruit accessions and obtained a total of 84591 non‐redundancy SVs using the Svimmer software (https://github.com/DecodeGenetics/svimmer). The results showed that the SVs were unevenly distributed on 29 chromosomes, and the SV density on Chr16 was the lowest (Figure S1a, Supporting Information). For each chromosome, the SV distribution is also uneven with some hotspot regions. Using 400Kb as a window and 200Kb as a step, the SV statistics of the whole genome were carried out (Figure S1b, Supporting Information), and 161 SV hot spots were identified (Data S4, Supporting Information), indicating that multiple, independent SVs have arisen in these regions. We found one hotspot region on Chr27 that contains 12 LRR‐RK genes (Figure S1c, Supporting Information), which play a key role in signal recognition and in response to environmental stimulus. These findings are consistent with the view that SV hotspot regions might undergo stronger environmental selection in contrast to the SVs located in other genomic regions.^[^ ^58^ ^]^

It is well known that the pangenome provides an opportunity to detect structural variations using short reads. So we constructed a graph‐based pangenome for kiwifruit by integrating all detected structural variations into the Hongyang.hap1 genome based on coordinates (Figure 4e). We believe it will serve as a crucial reference genome for future population studies on kiwifruit, particularly in utilizing structural variations for genotyping.

### 2.4 Overview of Structural Variations Associated with Fruit Colors

Kiwifruit displays a rich diversity of fruit colors including green, yellow, red, purple, and even the combinations. Fruit color is an important agronomic trait determining its commercial values. Generally, three main pigments including chlorophyll, carotenoid, and flavonoids contribute to fresh coloration in kiwifruit.^[^ ^6^ ^]^ No matter whether in RF, YF, or GF cultivars, fruits appear to be green at the early developmental stage, resulting from chlorophyll accumulation. Its red/purple coloration is due to the biosynthesis of anthocyanins at a later stage during fruit development.^[^ ^6^, ^59^, ^60^, ^61^, ^62^, ^63^, ^64^, ^65^ ^]^ The display of the yellow color from carotenoids is dependent on the degreening (chlorophyll degradation) during the ripening process.^[^ ^6^, ^66^ ^]^ Several studies showed the degradation of chlorophyll in RF and YF was partially caused by the high expression of *Staygreen 2* (*SGR2*) encoding a key and rate‐limiting enzyme for chlorophyll degradation in the ripening stage of kiwifruit,^[^ ^3^, ^6^, ^55^, ^67^, ^68^ ^]^ suggesting the chlorophyll degradation might play a key role in fruit coloration of kiwifruit.

In this study, we identified a total of 149 and 108 structural variants (SVs) associated with the anthocyanin accumulation and chlorophyll degradation pathway, respectively (Data S4 and S5, Supporting Information). Among the 149 SVs identified in the anthocyanin pathway, 14 are red‐fleshed group specific (presence in the red‐fleshed group but absence in other groups, and vice versa) (Data S4 and Figure S2, Supporting Information). Specifically, these unique variations involve genes such as *FLS*, *F3H*, and *F3'H*, with one located upstream, two downstream, and 16 in intergenic regions of the individual functional genes. Based on the transcriptomic analysis, most of these genes showed very low expression levels in fruit (Data S5, Supporting Information). Only one gene *F3H* (Achv4p01g000245) was substantially expressed in GF “Biyu” but was downregulated in RF “Hongyang” (Data S5, Supporting Information), suggesting that *F3H* is not the key gene affecting the anthocyanin accumulation in fruits of RF cultivars. Unfortunately, no RF‐specific SV was identified in the characterized genes, such as *AcMYB110*, *MYB27*, and *AcMYB123*, which have been demonstrated to regulate anthocyanin accumulation in kiwifruit.^[^ ^61^, ^63^, ^65^ ^]^

**Figure 5:**
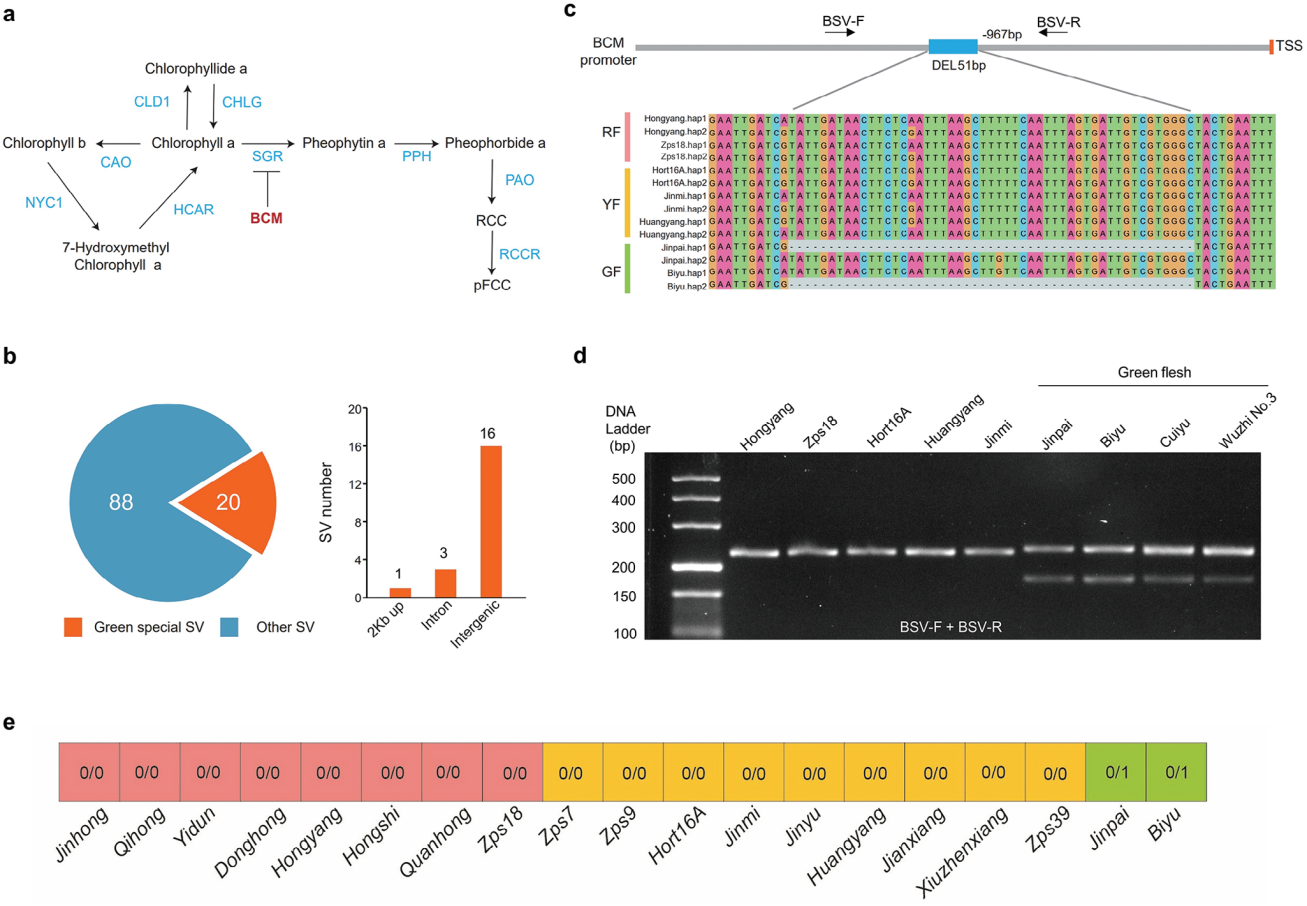
Identification of SVs related to chlorophyll degradation a) The diagram shows the metabolites and enzymes involved in chlorophyll catabolism. b) The piechat represents the number of green‐fleshed (GF) group specific SVs (highlighted in the orange) and the barplots indicated the location of GF specific SVs in the different regions of the corresponding genes. 2 Kb up (2000bp upstream of ATG) indicates promoter. c) A SV located at the promoter of *BCM* was specific in GF group. BSV‐F and BSV‐R indicate the location of a pair of primers for PCR verification of the SV. d) PCR verification of the SV in promoter of *BCM* by using BSV‐F and BSV‐R. e) Genotype of the SV of BCM promoter in other 12 varieties using the graph pangenome. The colors in the squares represent flesh color groups, and the numbers indicate genotypes.

Among the 108 SVs identified in the chlorophyll degradation pathway (**Figure** **5**a), 20 are specific in green‐fleshed cultivars (presence in the green‐fleshed group but absence in other groups, vice versa) (Figure 5b; Data S6, Supporting Information). Some green‐fleshed specific SVs were related to the key genes of chlorophyll catabolism such as *SGR*, *SGRL*, and *NYC1* (Data S6, Supporting Information). However, most SVs are located in intergenic regions and introns (Figure 5b) and these genes didn't show obviously different expressions between RF “Hongyang” and GF “Biyu” (Data S6, Supporting Information). Only one SV (51 bp deletion, Chr24:16 831 185) located in the promoter of *BCM* (*Balance of Chlorophyll Metabolism*) homologous gene (*AcBCM*) was detected in GF group “Biyu” (hap2) and “Jinpai” (hap1) (Figure 5a,c) and the expression levels of *AcBCM* is significantly different between Hongyang and Biyu (Data S6, Supporting Information). Besides, we aligned the resequencing data of the other 12 varieties from the RF and YF groups to the graph pangenome of kiwifruit for SV detection, and the results revealed the absence of the SV (Chr24:16 831 185) across all these 12 varieties (Figure 5e), reinforcing that this variation is specific to the GF group. Sequence analysis revealed that this SV is a Helitron type transposalbe element (TE). Furthermore, PCR analysis using a pair of primers (BSV‐F + BSV‐R, indicated in Figure 5c) showed that one band for RF and YF cultivars but two bands for GF were detected, respectively (Figure 5d). The Sanger sequencing of the two bands confirmed the existence of the tested SV. In Arabidopsis, overexpression of *BCM* causes chlorophyll accumulation by destabilizing SGR1.^[^ ^34^ ^]^ Therefore, we speculate that the SV‐induced high expression of *BCM* in “Biyu” and “Jinpai” might cause chlorophyll accumulation (green‐fleshed) by inhibiting SGR in kiwifruit. The green‐flesh cultivars of *A. chinensis* were very limited. We also found two additional green‐flesh cultivars of *A. chinensis* (Cuiyu and Wuzhi No.3), which are tetraploidy, and checked the SV by PCR. As shown in the Figure 5d, both tetraploidy green‐flesh cultivars also contain the variation in the promoter of the *BCM* gene, which was confirmed by Sanger sequencing. These results further confirmed the correlation of this variation with green‐flesh phenotype in *A. chinensis*.

### 2.5 The SV in *AcBCM* Promoter in GF Cultivars Increases *AcBCM* Expression and Chlorophyll Accumulation

**Figure 6:**
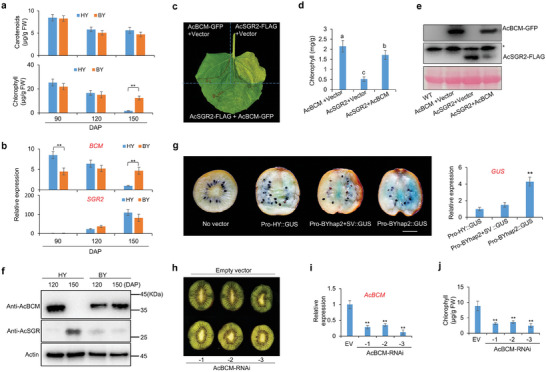
The SV‐induced high expression of AcBCM enhances chlorophyll accumulation by destabilizing AcSGR a) The quantitative measurement of carotenoids and chlorophyll contents in different developmental stages of ‘Hongyang (HY)’ and ‘Biyu (BY)’ fruits. DAP: Days after pollination. b) The qRT‐PCR analysis of *BCM* and *SGR2* expression in different developmental stages of HY and BY fruits. c–e) The phenotypes c), chlorophyll contents d), and western blot analysis e) of tobacco leaves transiently expressing AcBCM‐GFP, AcSGR2‐FLAG, and the combination. “*” indicates a non‐specific band. Data shown are averages±SD; *n* > 3. Significant differences were calculated by using two‐way ANOVA and Tukey's multiple comparison test and indicated with (a, b and c) (*p* < 0.01). g) The GUS staining of Hongyang 145 DAP fruits transiently expressing Pro‐HY::GUS, Pro‐BYhap2+SV::GUS, Pro‐BYhap2:GUS, and no vector as control. The qRT‐PCR analysis of *GUS* expression in the fruits injected with three vectors. Mean±SD values were calculated from 6 biological replicates. “**” indicates significant differences at *p* < 0.001. h–j) The phenotypes h), qRT‐PCR analysis of *AcBCM* i), and chlorophyll contents j) of 140 DAP green‐fleshed fruits transiently expressing AcBCM‐RNAi and empty vector (as control). Mean±SD values were calculated from 6 biological replicates. “**” indicates significant differences at *p* < 0.001.

The RF cultivar “Hongyang” (HY) and GF cultivar “Biyu” (BY) were used to study the possible role of the SV. The carotenoid contents showed no significant difference between HY and BY (**Figure** **6**a). The chlorophyll contents of 90‐ and 120‐days‐after‐pollination (‐DAP) fruits also showed no difference between HY and BY (Figure 6a). However, in 150 DAP, BY had much higher chlorophyll contents than HY (Figure 6a), which can be considered as a “staygreen” phenotype in the ripe stage of BY. The qRT‐PCR indicated that the *AcBCM* expression level in 150‐DAP fruits of HY was significantly lower than that in BY (Figure 6b). By transiently expressing BY *SGR2* (*AcSGR2*), *AcBCM*, and the combination of both genes in tobacco leaves, we revealed that overexpression of *AcBCM* could inhibit the *AcSGR2*‐induced chlorophyll degradation (Figure 6c,d). Western blot analysis also confirmed that *AcBCM* overexpression could decrease the protein abundance of AcSGR2 (Figure 6e), suggesting that *AcBCM* has the same function as *BCM* in Arabidopsis.^[^ ^34^ ^]^ Besides, we made the antibodies recognizing the endogenous AcSGR2 and AcBCM proteins, respectively. The western blots confirmed that, in the 150 DAP, the BCM protein levels in BY were much higher than that in HY (Figure 6f), which is consistent with qRT‐PCR results (Figure 6b). The SGR protein levels in BY were remarkably lower than that in HY (Figure 6f), which might result from the BCM‐induced degradation of SGR2 because the mRNA levels of *SGR2* in BY were just slightly lower than that in HY (Figure 6b). Moreover, *SGR2* mRNA levels in 120 DAP were much lower than that in 150 DAP of BY (Figure 6b) but its protein levels in 120 DAP were slightly higher than that in 150 DAP (Figure 6f), which might be caused by higher abundance of AcBCM in 150 DAP (Figure 6f). These results indicated the AcBCM‐induced AcSGR2 protein degradation occurred and caused chlorophyll accumulation in the late developmental stage of BY.

To determine whether the SV affects *AcBCM* gene expression at the late developmental stage, we used the *AcBCM* promoter from HY (Pro‐HY) and a promoter from BY hap2 (Pro‐BYhap2) without the 51 bp fragment, to drive *GUS* expression. The *Agrobacteria* containing both vectors were injected in 145 DAP fruits of HY and GUS staining indicated that Pro‐BYhap2 has significantly higher transcription activity than Pro‐HY (Figure 6g) and qRT‐PCR analysis of *GUS* gene also confirmed the results of GUS staining (Figure 6g). Moreover, we added the SV (51 bp) to the Pro‐BYhap2 to construct a mutant promoter Pro‐BYhap2+SV, which is similar to Pro‐BYhap1. GUS staining and qRT‐PCR indicated that the transcription activity of Pro‐BYhap2+SV is similar to Pro‐HY but much lower than Pro‐BYhap2 (Figure 6g). These results indicated that the SV (51 bp deletion) in the *BCM* promoter leads to its higher expression in the late developmental stage of BY fruits. Besides, we constructed an RNAi vector to silence *BCM* expression. The 140‐DAP fruits of the GF cultivar were infiltrated with *Agrobacteria* containing AcBCM‐RNAi vector or empty vector (as control). The fruits injected with the AcBCM‐RNAi vector showed less green color than that with the empty vector (Figure 6h). The qRT‐PCR and quantitative measurements of chlorophyll contents indicated that *AcBCM* mRNA levels (Figure 6i) and chlorophyll contents (Figure 6j) were significantly decreased in the fruits of AcBCM‐RNAi, compared with that of empty vector. These results indicated that SV‐induced high expression of *AcBCM* contributes to chlorophyll accumulation in BY.

## 3 Discussion

In this study, we have *de novo* assembled 12 chromosome‐scale and haploid‐resolved haplotypes for 6 representative accessions of *A. chinensis*, and then constructed a high‐confidence pangenome by using the Hongyang T2T genome (v4.0) as a reference.^[^ ^31^ ^]^ The pangenome is 113.4 Mb larger than the reference genome and includes 3961 genes not found in the reference genome. Therefore, these 14 representative high‐quality genome assemblies have revealed much greater genetic diversity than a single reference genome can represent. The pangenome makes it possible to detect SVs using the short reads of next‐generation sequencing (NGS) at the population level in kiwifruit, which will promote the development of kiwifruit population genomics, especially in exploring structural variations potentially associated with key agricultural traits.

The pan‐genome is expected to represent the majority of genetic variations within a species, which depends on the quantity and diversity of the samples.^[^ ^69^ ^]^ In this study, the selected samples were sourced extensively, representing the vast majority of commercially grown diploid kiwifruit cultivars currently available. The stabilization of the pangenome size at ≈11 indicates that we have successfully captured a significant portion of the genetic diversity present in the tested species. Kiwifruit (*A. chinensis*) exhibits a remarkably higher number of SVs compared to other species with alike genome sizes, such as Medicago,^[^ ^70^ ^]^ which can be attributed to its dioecious nature and high heterozygosity, leading to extensive genetic diversity. Furthermore, the much shorter domestication history (about 100 years) could also contribute to the high diversity of *Actinidia* species. Of course, we can't exclude the possibility that the small sample size in other studies would cause the undervaluation of biodiversity. The methodology employed in this study provides clues for further investigations aiming to discover the entire genetic diversity by incorporating genome assemblies from a broader range of accessions in *A. chinensis*. Additionally, we found that dispensable genes, especially the shell genes, exhibit a higher evolutionary rate than core genes (Figure 2i; Table S7, Supporting Information), suggesting that they may be subject to stronger environmental selective pressures. It is worth noting that dispensable genes are significantly enriched in the sucrose and anthocyanin metabolic pathways (Table S9, Supporting Information), indicating a rich diversity in the quality of different kiwifruit accessions.

Fruit color is one of the key appearance features determining the commercial value. Chlorophyll accumulation in the late developmental stage leads to green flesh in GF but its degradation results in the degreening of YF or RF. The previous studies showed that the fruit‐specific *AcSGR2* gene displays higher expression in YF and RF than in GF, which contributes to the degreening in YF fruit.^[^ ^3^, ^6^, ^67^, ^68^ ^]^ A transcription factor AcHZP45 suppresses chlorophyll biosynthesis and enhances chlorophyll degradation by activating *AcSGR1* and *AcSGR2*.^[^ ^55^ ^]^ Some studies indicated that the downregulation of the *SGR* gene is not a sufficient factor in determining the green flesh and other factors, such as the phytohormone, are also involved.^[^ ^3^ ^]^

Fruits of *A. chinensis* show the greatest diversity in colorations. Therefore, our pangenome of *A. chinensis* should facilitate the identification of SVs controlling fruit color. In this study, we discovered 20 SVs associated with the structural genes involved in chlorophyll degradation and identified an SV exclusively present in the promoter of the *AcBCM* gene in GF cultivars (Biyu and Jipai). The green‐flesh cultivars of *Actinidia chinensis* were very limited. We found two additional tetraploidy green‐flesh cultivars of *A. chinensis* (Cuiyu and Wuzhi No.3)(Figure 5d). PCR analysis and Sanger sequencing also showed the presence of the SV in their genomes, which further strengthened the correlation of SV with green‐flesh phenotype in *A. chinensis*.

This SV was demonstrated to cause the higher expression of *AcBCM* in the late developmental stage of fruit (Figure 6). Silencing *AcBCM* caused de‐greening of Biyu (Figure 6). In Arabidopsis, *BCM* overexpression caused chlorophyll accumulation by inducing the degradation of SGR1 proteins.^[^ ^34^ ^]^ Our work also indicated that overexpression of *AcBCM* destabilized AcSGR2 proteins and inhibited AcSGR2‐induced chlorophyll degradation (Figure 6c,d). The Western blot analysis also confirmed that AcBCM proteins are much higher but AcSGR2 proteins are much lower in 150 DAP of BY, as compared with that of HY (Figure 6e). Based on these results, we speculate that, in YF and RF cultivars of *A. chinensis*, *AcBCM* was significantly downregulated but *AcSGR2* was upregulated during the ripening process, which led to protein accumulation of AcSGR2 and chlorophyll degradation. In GF cultivars, *AcSGR2* was upregulated as well, but the SV‐mediated high expression of *AcBCM* destabilized AcSGR2 at the protein level, consequently inhibiting the chlorophyll degradation. These results indicated that the AcBCM‐mediated post‐translational regulation of *AcSGR2*, which is neglected by the previous studies focusing on transcriptional regulation, also plays an important role in fruit coloration in kiwifruit.

As for the question how the SV affects *BCM* expression, we discovered there are a few *cis*‐elements existing in the deletion sequence by using the plant transcription factor database (PlantTFDB v5.0). We speculated that some transcription factor may recognize the *cis* element in the variation to suppress the *BCM* expression. The deletion of the 51 bp in the green‐flesh cultivar might abolish the suppression from the transcription factor. Of course, we can't exclude another possibility that the deletion of 51 bp might create a novel *cis*‐element for promoting the BCM expression. The exact mechanisms require further investigation.

Collectively, we have generated a graph‐based pangenome dataset for *A. chinensis* and other *Actinidia* species. Our work disclosed a resource of SVs for in‐depth functional genomics studies. We characterized an SV in the promoter of the *AcBCM* gene, which affects *AcBCM* expression and causes chlorophyll accumulation in the green‐flesh fruits by post‐translationally regulating AcSGR2. Our work discovered the first SV mediating fruit degreening process and revealed the previously unknown mechanisms of green flesh formation in kiwifruit.

## 4 Experimental Section

### SNP Calling and Phylogenetic Analyses

Fresh, young, and healthy leaves were collected from 19 different kiwifruit accessions at the Germplasm Resources Nursery of Anhui Agriculture University for whole genome resequencing. The Illumina short reads were first aligned to the HY4P reference genome^[^ ^31^ ^]^ using BWA v0.7.17.^[^ ^35^ ^]^ Next, non‐unique and unmapped reads were filtered out using SAMtools,^[^ ^36^ ^]^ and duplicated reads were removed with the Picard package (v1.87). Finally, SNPs were identified using the Genome Analysis Toolkit (GATK) package.^[^ ^37^ ^]^

Only SNPs that satisfied the conditions of a missing data rate of less than 15% and a minor allele count higher than three were used for phylogenetic analyses. A phylogenetic tree of the 19 accessions was constructed using the Phylip software (version 3.696) with the neighbor‐joining (NJ) method^[^ ^38^ ^]^ and “White”^[^ ^39^ ^]^ as the outgroup. The bootstrap process was replicated 1000 times.

### Sample Preparation and Sequencing

Based on the phylogenetic tree, seven representative accessions were chosen for PacBio HiFi sequencing, including those with three distinct flesh colors: red (“Hongyang” and “Zps18”), yellow (“Hort16A”, “Jinmi” and “Huangyang”), and green (“Biyu” and “Jinpai”). Genomic DNAs were extracted from these samples using the CTAB method,^[^ ^40^ ^]^ and then subjected to construct 150‐bp paired‐end libraries for Illumina sequencing on an Illumina Novoseq 6000 platform. In addition, a standard SMRTbell library was constructed using 50 µg of the six genomic DNA for PacBio HiFi sequencing, following the manufacturer's instructions with the SMRTbell Express Template Prep Kit 2.0. The final PacBio library was sequenced using a PacBio Sequel II system (Pacific Biosciences, CA, USA).

### Genome Assembly and Annotation

Initially, the primary haplotype (HY4P) of the T2T genome Hongyang 4.0^[^ ^31^ ^]^ was selected as the reference genome. The HiFi reads from six representative accessions were then *de novo* assembled into two haplotype‐resolved contigs using hifasm v0.16.1 software.^[^ ^41^ ^]^ Subsequently, the HiFi contigs were anchored and oriented to chromosomes using a reference‐guided strategy to achieve a chromosome‐level assembly, which was performed with quarTeT software (http://www.atcgn.com:8080/quarTeT/home.html).^[^ ^42^ ^]^ A comprehensive evaluation approach to assess the quality of genome assembly was used. First, the wholeness of genome assembly was evaluated by the BUSCO assessment,^[^ ^43^ ^]^ which included 1614 genes in the Embryophyta OrthoDB v10 dataset (https://www.orthodb.org). Second, the continuity of genome assembly was accessed by the contigs N50 values and the LAI scores.^[^ ^44^ ^]^ Third, the accuracy of genome assembly was estimated with mapping the short reads were to calculate the mapping rate using BWA v0.7.17.^[^ ^35^ ^]^

In this study, the braker2 pipelines^[^ ^45^ ^]^ were used to predict protein‐coding genes for each genome assembly, and 36 RNA‐seq datasets used for Hongyang v4.0 annotation^[^ ^31^ ^]^ were also employed to aid in the gene prediction process. Only those genes that contained both start and stop codons and were at least 100 amino acids (aa) in length were reserved. The predicted genes were annotated by conducting BLASTP (version 2.6) and diamond (version 0.9.23) searches of the encoded protein sequences against several protein databases, including NCBI, Swiss‐Prot, KIR (http://kir.atcgn.com/), TAIR (https://www.arabidopsis.org/), and KGD (http://kiwifruitgenome.org/), using an *E*‐value threshold of 1*e*‐5. Then, the Blast2GO local pipeline (version 3.2) to analyze gene ontology (GO) terms to each protein‐coding gene was used. To identify domains or motifs within the protein sequences, an InterProScan software^[^ ^46^ ^]^ and searched against the Pfam database were utilized.

### Pan‐Genome Construction

To construct the family‐based pangenome, a gene family clustering to estimate the core and dispensable gene sets was used. First, the OrthoFinder^[^ ^47^ ^]^ was used to cluster genes into families, and then the gene families that were shared by all 14 assemblies were extracted as the core gene clusters. Gene families that were missing in one or more kiwifruit accessions were defined as dispensable gene clusters. The dispensable gene clusters were further categorized into softcore (genes in those clusters present in more than 80% assemblies), cloud (genes in those clusters present in only one assembly), and shell (genes in the rest clusters) clusters.

### Detection of Genomic Variations

The genome sequences of other 13 haplotype assemblies were aligned to the HY4P reference genome using the minimap2 software^[^ ^48^ ^]^ with the default parameters. And then the aligned results were parsed using SyRI software^[^ ^49^ ^]^ to identify genomic variations, including presence/absence variations (PAVs) (in terms of large InDels that are ≥50 bp in size), inversions, and translocations. Subsequently, SVs (including presence/absence variations) from 13 assemblies were merged into a non‐redundant set of structural variations using swimmer with parameters ′–ids –max_distance 50 –max_size_difference 20″. The variation effect of all SVs was annotated using the snpEFF software. To identify structural variation (SV) hotspots, a statistical analysis of SVs on the chromosome using a window size of 400 kb and a step size of 200 kb was conducted. Subsequently, an intervals with SV number exceeding 80 and merged contiguous widows as hotspot regions were extracted.

### RNA Sequencing and Data Analysis

Total RNA was extracted from fruit samples of “Hongyang” and “Biyu” collected at 100, 120, and 150 days after pollination. Each sample comprised three biological replicates. Subsequently, mRNA libraries were constructed and sequenced using the NovoSeq 600 platform. The clean reads were then mapped to the HY4P^[^ ^31^ ^]^ reference genome using HISAT2^[^ ^50^ ^]^ software with default parameters. Transcripts per million (TPM) values were calculated using featureCounts.^[^ ^51^ ^]^ The differential expression analysis was performed using the DESeq2 package^[^ ^52^ ^]^ in R and the the screening criteria were set as ′|log_2_(fold change)| >1 and the *p* value<0.05″.

### Structural Variation Analysis of Anthocyanin and Chlorophyll Pathways

To identify members of the gene family involved in anthocyanin biosynthesis and chlorophyll degradation metabolic pathways, BLASTP searches were performed against the HY4P protein sequences using the full‐length protein sequences related to these pathways in Arabidopsis, sweet orange, apple, tomato, and wine grape downloaded from NCBI as queries. Only these with *E*‐values < 1×10^−10^ and with a high alignment score were selected as candidate genes. And then, SVs related to these genes were extracted based on their positions for further analysis.

### Analysis of Genome Synteny

The MUMmer4 tool was used to perform genome‐wide comparisons between the 14 assemblies, with the parameters (–maxmatch, ‐c 500, ‐b 200, and ‐l 100).^[^ ^53^ ^]^ The resulting alignments were then screened by using a delta‐filter with the parameters (‐m, ‐i 90, and ‐l 100). Finally, dot plot representations were generated to visualize the genome synteny using Mummerplot.

### Resolution of Metabolite Profiling

Metabolome profiles were generated using a widely targeted metabolomics method. Initially, the fruit samples used for metabolomics analysis of these seven kiwifruit accessions were all harvested at 140 days after pollination (DAP) and stored at room temperature for 10 days. Then, the flesh samples were freeze–dried and homogenized through a mixer mill (MM 400, Retsch) with a zirconia bead for 1.5 min at 30 Hz. Then, 100 mg of tissue powders were mixed with 1.2 mL of 70% methanol solution at 4 °C overnight, followed by centrifugation at 12 000 rpm for 10 min. The resulting supernatants were collected, filtered through a 0.22 µm pore size membrane (SCAA‐104, ANPEL, Shanghai, China), and analyzed by UPLC‐MS/MS (UPLC, SHIMADZU Nexera X2; MS, Applied Biosystems 4500 Q TRAP). Metabolite quantification was performed by multiple reaction monitoring (MRM) methods.

### Transient Expression, GUS Staining, and RNA Interference

The transient expression in fruit was performed under the improvement of the protocol.^[^ ^54^ ^]^ The construct‐carrying A. tumefaciens was suspended in MMA solution (1/2 MS, 10 mm MES, 150 µm Acetosyringone), infiltrated into the pulp around the fruit, and infiltrated into the core from the end. Then it was placed on wet filter paper to moisturize and stored in a 25 °C tissue incubator for 3 days. The fruits were sectioned and immersed in the GUS staining solution, kept in a vacuum pump at 0.8 MPa for 10 min, and then placed in a 37 °C incubator for staining and decolorization. For RNA interference, 140 DAP fruits of GF cultivars were injected as described previously^[^ ^54^ ^]^ with *A. tumefaciens* EHA105 containing AcBCB‐RNAi vector or empty vector. The phenotype observation, qRT‐PCR, and chlorophyll content measurements were performed 6 days after infiltration.

### Western Blot Analysis

The tobacco leaves transiently expressing *AcBCM‐GFP* and *AcSGR2*‐*FLAG* or fruit samples of Hongyang and Biyu were frozen and fully ground in a mortar with liquid nitrogen. After weighing in the EP tube, the total proteins were dissolved with four volumes of 2×loading sample buffer (8 m Urea, 100 mm Tris‐HCl pH = 6.8, 4%SDS, 0.2%BPB, 20%Glycerol, 2% TritonX‐100, 5%β‐Me). Total proteins were denatured at 100 °C for 10 min and separated by SDS‐PAGE, transferred to a PVDF (0.22 µm for AcSGR and 0.45 µm for AcBCM) blotting membrane for immunization and detection. For making Anti‐AcBCM and Anti‐SGR2 antibodies, two peptides from AcBCM and AcSGR, respectively, were synthesized and injected into rabbits for producing polyclonal antibodies, which were performed by GenScript Biotech Corporation. Anti‐FLAG antibodies (ABT2010) were purchased from Abbkine Scientific Co.,Ltd. The antibodies against GFP (300 943) and HRP‐conjugated goat anti‐rabibit secondary antibodies (ZB‐5301) were purchased from ZEN BIO and ZSGB‐BIO, respectively.

### Chlorophyll and Carotenoid Measurement

Extraction and measurement of chlorophyll and carotenoid was conducted according to previously reported methods.^[^ ^55^ ^]^ All steps were performed at 4 °C in the dark. ≈0.6 g of kiwifruit flesh powder was weighed and extracted with 2 mL 80% acetone (v/v) for 1 h. The mixture was then centrifuged at 12 000 g for 10 mi and the supernatants were transferred to a new tube stored in ice. The extraction process was repeated three times until the powder was completely whitened. Then, the supernatant of the three extractions was mixed and centrifuged at 12 000 g for 10 min and involved measuring the absorbance at 663, 645, and 470 nm using UV‐1000 Spectrophotometer (AOE Lab).

### Quantitative RT‐PCR

Total RNA was isolated using Universal Plant Total RNA Isolation Kit (Vazyme), and cDNA synthesis was performed with 1st Strand cDNA Synthesis Kit (Yeasen). Before running Real‐time quantitative PCR, the PCR products were separated on 2% agarose gel and sequenced to confirm the specificity and accuracy of the PCR reaction. The quantitative PCR was conducted using qPCR SYBR Green Master Mix (Yeasen). All PCR reactions were performed on the Bio‐Rad CFX96 Real‐time PCR System with at least three biological replicates. The transcript expression levels were quantified by the 2^−ΔΔCT^ method^[^ ^56^ ^]^ using *AcUBI* as the endogenous control.

## Conflict of Interest

The authors declare no conflict of interest.

## Author Contributions

Y.W., and P.L. contributed equally to this work. Y.L., S.W., and J.Y. planned and designed the research. J.Y., Y.W., P.L., F.Z., S. Z., Y.W., Y.L., H.W., W.R., L.W., Y.Y., R.W., and P.Z. analyzed data. P.L., Y.Z., Y.H., and Y. W. performed the experiments. S.W., J.Y., and Y.W. wrote the manuscript. S.W. and Y.L. revised the manuscript.

## Supporting information

Supporting Information

Supporting Information

## Data Availability

The genome sequencing data have been deposited at the Sequence Read Archive database in NCBI and the accession number is PRJNA979323. The genome assemblies have been deposited at the Genome Warehouse in NGDC and the accession number is PRJCA026075. The data and materials included in the study are available from the corresponding authors upon request.

## References

[1] J. Giovannoni , C. Nguyen , B. Ampofo , S. Zhong , Z. Fei , Annu. Rev. Plant Biol. 2017, 68, 61. 28226232 10.1146/annurev-arplant-042916-040906

[2] T. Martins , A. N. Barros , E. Rosa , L. Antunes , Molecules 2023, 28, 5344. 37513218 10.3390/molecules28145344 PMC10384064

[3] S. M. Pilkington , M. Montefiori , P. E. Jameson , A. C. Allan , Planta 2012, 236, 1615. 22843245 10.1007/s00425-012-1723-x

[4] S. Huang , J. Ding , D. Deng , W. Tang , H. Sun , D. Liu , L. Zhang , X. Niu , X. Zhang , M. Meng , J. Yu , J. Liu , Y. Han , W. Shi , D. Zhang , S. Cao , Z. Wei , Y. Cui , Y. Xia , H. Zeng , K. Bao , L. Lin , Y. Min , H. Zhang , M. Miao , X. Tang , Y. Zhu , Y. Sui , G. Li , H. Sun , Nat. Commun. 2013, 4, 2640. 24136039 10.1038/ncomms3640 PMC4089393

[5] X. Yuan , H. Zheng , J. Fan , F. Liu , J. Li , C. Zhong , Q. Zhang , Foods 2022, 12, 108. 36613324 10.3390/foods12010108 PMC9818353

[6] Y. Liu , B. Zhou , Y. Qi , X. Chen , C. Liu , Z. Liu , X. Ren , Front Plant Sci 2017, 8, 1507. 28919902 10.3389/fpls.2017.01507 PMC5586210

[7] D. Xie , Y. Xu , J. Wang , W. Liu , Q. Zhou , S. Luo , W. Huang , X. He , Q. Li , Q. Peng , X. Yang , J. Yuan , J. Yu , X. Wang , W. J. Lucas , S. Huang , B. Jiang , Z. Zhang , Nat. Commun. 2019, 10, 5158. 31727887 10.1038/s41467-019-13185-3 PMC6856369

[8] T. Lin , G. Zhu , J. Zhang , X. Xu , Q. Yu , Z. Zheng , Z. Zhang , Y. Lun , S. Li , X. Wang , Z. Huang , J. Li , C. Zhang , T. Wang , Y. Zhang , A. Wang , Y. Zhang , K. Lin , C. Li , G. Xiong , Y. Xue , A. Mazzucato , M. Causse , Z. Fei , J. J. Giovannoni , R. T. Chetelat , D. Zamir , T. Städler , J. Li , Z. Ye , et al., Nat. Genet. 2014, 46, 1220. 25305757 10.1038/ng.3117

[9] T. Akagi , I. M. Henry , H. Ohtani , T. Morimoto , K. Beppu , I. Kataoka , R. Tao , Plant Cell 2018, 30, 780. 29626069 10.1105/tpc.17.00787 PMC5969274

[10] A. A. Golicz , J. Batley , D. Edwards , Plant Biotechnol J 2016, 14, 1099. 26593040 10.1111/pbi.12499 PMC11388911

[11] J. Ebler , P. Ebert , W. E. Clarke , T. Rausch , P. A. Audano , T. Houwaart , Y. Mao , J. O. Korbel , E. E. Eichler , M. C. Zody , A. T. Dilthey , T. Marschall , Nat. Genet. 2022, 54, 518. 35410384 10.1038/s41588-022-01043-w PMC9005351

[12] H. Tettelin , V. Masignani , M. J. Cieslewicz , C. Donati , D. Medini , N. L. Ward , S. V. Angiuoli , J. Crabtree , A. L. Jones , A. S. Durkin , R. T. DeBoy , T. M. Davidsen , M. Mora , M. Scarselli , I. Margarit y Ros , J. D. Peterson , C. R. Hauser , J. P. Sundaram , W. C. Nelson , R. Madupu , L. M. Brinkac , R. J. Dodson , M. J. Rosovitz , S. A. Sullivan , S. C. Daugherty , D. H. Haft , J. Selengut , M. L. Gwinn , L. Zhou , N. Zafar , Proc Natl Acad Sci 2005, 102, 13950. 16172379 10.1073/pnas.0506758102 PMC1216834

[13] Y. Zhou , Z. Zhang , Z. Bao , H. Li , Y. Lyu , Y. Zan , Y. Wu , L. Cheng , Y. Fang , K. Wu , J. Zhang , H. Lyu , T. Lin , Q. Gao , S. Saha , L. Mueller , Z. Fei , T. Städler , S. Xu , Z. Zhang , D. Speed , S. Huang , Nature 2022, 606, 527. 35676474 10.1038/s41586-022-04808-9 PMC9200638

[14] L. Gao , I. Gonda , H. Sun , Q. Ma , K. Bao , D. M. Tieman , E. A. Burzynski‐Chang , T. L. Fish , K. A. Stromberg , G. L. Sacks , T. W. Thannhauser , M. R. Foolad , M. J. Diez , J. Blanca , J. Canizares , Y. Xu , E. van der Knaap , S. Huang , H. J. Klee , J. J. Giovannoni , Z. Fei , Nat. Genet. 2019, 51, 1044. 31086351 10.1038/s41588-019-0410-2

[15] N. Li , Q. He , J. Wang , B. Wang , J. Zhao , S. Huang , T. Yang , Y. Tang , S. Yang , P. Aisimutuola , R. Xu , J. Hu , C. Jia , K. Ma , Z. Li , F. Jiang , J. Gao , H. Lan , Y. Zhou , X. Zhang , S. Huang , Z. Fei , H. Wang , H. Li , Q. Yu , Nat. Genet. 2023, 55, 852. 37024581 10.1038/s41588-023-01340-y PMC10181942

[16] J. M. Song , Z. Guan , J. Hu , C. Guo , Z. Yang , S. Wang , D. Liu , B. Wang , S. Lu , R. Zhou , W. Z. Xie , Nat Plants 2020, 6, 34. 31932676 10.1038/s41477-019-0577-7 PMC6965005

[17] Q. Qiao , P. P. Edger , L. Xue , L. Qiong , J. Lu , Y. Zhang , Q. Cao , A. E. Yocca , A. E. Platts , S. J. Knapp , M. Van Montagu , Proc Natl Acad Sci 2021, 118, e2105431118. 34697247 10.1073/pnas.2105431118 PMC8609306

[18] H. Li , S. Wang , S. Chai , Z. Yang , Q. Zhang , H. Xin , Y. Xu , S. Lin , X. Chen , Z. Yao , Q. Yang , Z. Fei , S. Huang , Z. Zhang , Nat. Commun. 2022, 13, 682. 35115520 10.1038/s41467-022-28362-0 PMC8813957

[19] Y. Huang , J. He , Y. Xu , W. Zheng , S. Wang , P. Chen , B. Zeng , S. Yang , X. Jiang , Z. Liu , L. Wang , X. Wang , S. Liu , Z. Lu , Z. Liu , H. Yu , J. Yue , J. Gao , X. Zhou , C. Long , X. Zeng , Y.‐J. Guo , W.‐F. Zhang , Z. Xie , C. Li , Z. Ma , W. Jiao , F. Zhang , R. M. Larkin , R. R. Krueger , et al., Nat. Genet. 2023, 55, 1964. 37783780 10.1038/s41588-023-01516-6

[20] T. Wang , S. Duan , C. Xu , Y. Wang , X. Zhang , X. Xu , L. Chen , Z. Han , T. Wu , Nat. Commun. 2023, 14, 7377. 37968318 10.1038/s41467-023-43270-7 PMC10651928

[21] S. Chen , P. Wang , W. Kong , K. Chai , S. Zhang , J. Yu , Y. Wang , M. Jiang , W. Lei , X. Chen , W. Wang , Nat Plants 2023, 9, 1986. 38012346 10.1038/s41477-023-01565-z

[22] S. Wu , H. Sun , L. Gao , S. Branham , C. McGregor , S. S. Renner , Y. Xu , C. Kousik , W. P. Wechter , A. Levi , Z. Fei , Plant Biotechnol J 2023, 21, 1926. 37490004 10.1111/pbi.14120 PMC10502741

[23] J. Yue , J. Liu , R. Ban , W. Tang , L. Deng , Z. Fei , Y. Liu , Database 2015, 2015, bav113. 26656885 10.1093/database/bav113 PMC4674624

[24] H. Wu , T. Ma , M. Kang , F. Ai , J. Zhang , G. Dong , J. Liu , Hortic Res 2019, 6, 117. 31645971 10.1038/s41438-019-0202-y PMC6804796

[25] J. Yue , J. Liu , W. Tang , Y. Q. Wu , X. Tang , W. Li , Y. Yang , L. Wang , S. Huang , C. Fang , K. Zhao , Z. Fei , Y. Liu , Y. Zheng , Hortic Res 2020, 7, 117. 32821400 10.1038/s41438-020-0338-9 PMC7395147

[26] D. Scaglione , A. Fornasiero , C. Pinto , F. Cattonaro , A. Spadotto , R. Infante , C. Meneses , R. Messina , O. Lain , G. Cipriani , R. Testolin , Tree Genet. Genomes 2015, 11, 115.

[27] E. Popowski , S. J. Thomson , M. Knäbel , J. Tahir , R. N. Crowhurst , M. Davy , T. M. Foster , R. J. Schaffer , D. S. Tustin , A. C. Allan , J. McCallum , G3 (Bethesda) 2021, 11. 10.1093/g3journal/jkab142 PMC8495948 34009255

[28] Y. Liu , D. Li , Q. Zhang , C. Song , C. Zhong , X. Zhang , Y. Wang , X. Yao , Z. Wang , S. Zeng , Y. Wang , Y. Guo , S. Wang , X. Li , L. Li , C. Liu , H. C. McCann , W. He , Y. Niu , M. Chen , L. Du , J. Gong , P. M. Datson , E. Hilario , H. Huang , New Phytol 2017, 215, 877. 28543189 10.1111/nph.14607

[29] T. Akagi , S. M. Pilkington , E. Varkonyi‐Gasic , I. M. Henry , S. S. Sugano , M. Sonoda , A. Firl , M. A. McNeilage , M. J. Douglas , T. Wang , R. Rebstock , C. Voogd , P. Datson , A. C. Allan , K. Beppu , I. Kataoka , R. Tao , Nat Plants 2019, 5, 801. 31383971 10.1038/s41477-019-0489-6

[30] J. Yue , Q. Chen , S. Zhang , Y. Lin , W. Ren , B. Li , Y. Wu , Y. Wang , Y. Zhou , Y. Liu , Plant Biotechnol J 2024, 22, 287. 37905334 10.1111/pbi.14213 PMC10826980

[31] J. Yue , Q. Chen , Y. Wang , L. Zhang , C. Ye , X. Wang , S. Cao , Y. Lin , W. Huang , H. Xian , H. Qin , Y. Wang , S. Zhang , Y. Wu , S. Wang , Y. Yue , Y. Liu , Hortic Res 2023, 10, uhac264. 36778189 10.1093/hr/uhac264 PMC9909506

[32] X. Han , Y. Zhang , Q. Zhang , N. Ma , X. Liu , W. Tao , Z. Lou , C. Zhong , X. W. Deng , D. Li , H. He , Mol. Plant 2023, 16, 452. 36588343 10.1016/j.molp.2022.12.022

[33] Y. Wang , M. Dong , Y. Wu , F. Zhang , W. Ren , Y. Lin , Q. Chen , S. Zhang , J. Yue , Y. Liu , Mol Hortic 2023, 3, 4. 37789444 10.1186/s43897-023-00052-5 PMC10515003

[34] P. Wang , B. Grimm , Trends Plant Sci. 2021, 26, 484. 33422426 10.1016/j.tplants.2020.12.005

[35] H. Jo , G. Koh , Biomed Mater Eng 2015, 26, S1791. 26405948 10.3233/BME-151480

[36] H. Li , B. Handsaker , A. Wysoker , T. Fennell , J. Ruan , N. Homer , G. Marth , G. Abecasis , R. Durbin , Bioinformatics 2009, 25, 2078. 19505943 10.1093/bioinformatics/btp352 PMC2723002

[37] A. McKenna , M. Hanna , E. Banks , A. Sivachenko , K. Cibulskis , A. Kernytsky , K. Garimella , D. Altshuler , S. Gabriel , M. Daly , M. A. DePristo , Genome Res. 2010, 20, 1297. 20644199 10.1101/gr.107524.110 PMC2928508

[38] N. Saitou , M. Nei , Mol. Biol. Evol. 1987, 4, 406. 3447015 10.1093/oxfordjournals.molbev.a040454

[39] W. Tang , X. Sun , J. Yue , X. Tang , C. Jiao , Y. Yang , X. Niu , M. Miao , D. Zhang , S. Huang , W. Shi , Gigascience 2019, 8. 10.1093/gigascience/giz027 PMC6446220 30942870

[40] G. C. Allen , M. A. Flores‐Vergara , S. Krasynanski , S. Kumar , W. F. Thompson , Nat. Protoc. 2006, 1, 2320. 17406474 10.1038/nprot.2006.384

[41] H. Cheng , G. T. Concepcion , X. Feng , H. Zhang , H. Li , Nat. Methods 2021, 18, 170. 33526886 10.1038/s41592-020-01056-5 PMC7961889

[42] Y. Lin , C. Ye , X. Li , Q. Chen , Y. Wu , F. Zhang , R. Pan , S. Zhang , S. Chen , X. Wang , S. Cao , Y. Wang , Y. Yue , Y. Liu , J. Yue , Hortic Res 2023, 10, uhad127. 37560017 10.1093/hr/uhad127 PMC10407605

[43] M. Manni , M. R. Berkeley , M. Seppey , F. A. Simão , E. M. Zdobnov , Mol. Biol. Evol. 2021, 38, 4647. 34320186 10.1093/molbev/msab199 PMC8476166

[44] S. J. Ou , J. F. Chen , N. Jiang , Nucleic Acids Res. 2018, 46, e126. 30107434 10.1093/nar/gky730 PMC6265445

[45] K. J. Hoff , A. Lomsadze , M. Borodovsky , M. Stanke , Methods Mol Biol 2019, 1962, 65. 31020555 10.1007/978-1-4939-9173-0_5 PMC6635606

[46] N. Mulder , R. Apweiler , Methods Mol Biol 2007, 396, 59. 18025686 10.1007/978-1-59745-515-2_5

[47] D. M. Emms , S. Kelly , Genome Biol. 2019, 20, 238. 31727128 10.1186/s13059-019-1832-y PMC6857279

[48] H. Li , Bioinformatics 2018, 34, 3094. 29750242 10.1093/bioinformatics/bty191 PMC6137996

[49] M. Goel , H. Sun , W.‐B. Jiao , K. Schneeberger , Genome Biol. 2019, 20, 277. 31842948 10.1186/s13059-019-1911-0 PMC6913012

[50] D. Kim , J. M. Paggi , C. Park , C. Bennett , S. L. Salzberg , Nat. Biotechnol. 2019, 37, 907. 31375807 10.1038/s41587-019-0201-4 PMC7605509

[51] Y. Liao , G. K. Smyth , W. Shi , Bioinformatics 2014, 30, 923. 24227677 10.1093/bioinformatics/btt656

[52] M. I. Love , W. Huber , S. Anders , Genome Biol. 2014, 15, 550. 25516281 10.1186/s13059-014-0550-8 PMC4302049

[53] G. Marçais , A. L. Delcher , A. M. Phillippy , R. Coston , S. L. Salzberg , A. Zimin , PLoS Comput. Biol. 2018, 14, e1005944. 29373581 10.1371/journal.pcbi.1005944 PMC5802927

[54] S. Spolaore , L. Trainotti , G. Casadoro , J. Exp. Bot. 2001, 52, 845. 11413221 10.1093/jexbot/52.357.845

[55] Y. Wu , L. Wang , Y. Lin , X. Li , X. Liu , Z. Xu , B. Fu , W. Wang , A. Allan , M. Tu , X. Yin , J. Exp. Bot. 2023, 75, 204. 10.1093/jxb/erad361 37712824

[56] K. J. Livak , T. D. Schmittgen , Methods 2001, 25, 402. 11846609 10.1006/meth.2001.1262

[57] S. M. Pilkington , R. Crowhurst , E. Hilario , S. Nardozza , L. Fraser , Y. Peng , K. Gunaseelan , R. Simpson , J. Tahir , S. C. Deroles , K. Templeton , Z. Luo , M. Davy , C. Cheng , M. McNeilage , D. Scaglione , Y. Liu , Q. Zhang , P. Datson , N. De Silva , S. E. Gardiner , H. Bassett , D. Chagné , J. McCallum , H. Dzierzon , C. Deng , Y.‐Y. Wang , L. Barron , K. Manako , J. Bowen , BMC Genomics 2018, 19, 257. 29661190 10.1186/s12864-018-4656-3 PMC5902842

[58] W.‐B. Jiao , K. Schneeberger , Nat. Commun. 2020, 11, 989. 32080174 10.1038/s41467-020-14779-y PMC7033125

[59] M. Montefiori , R. V. Espley , D. Stevenson , J. Cooney , P. M. Datson , A. Saiz , R. G. Atkinson , R. P. Hellens , A. C. Allan , Plant J. 2011, 65, 106. 21175894 10.1111/j.1365-313X.2010.04409.x

[60] L. G. Fraser , A. G. Seal , M. Montefiori , T. K. McGhie , G. K. Tsang , P. M. Datson , E. Hilario , H. E. Marsh , J. K. Dunn , R. P. Hellens , K. M. Davies , M. A. McNeilage , H. N. D. Silva , A. C. Allan , BMC Genomics 2013, 14, 28. 23324587 10.1186/1471-2164-14-28 PMC3618344

[61] L. Wang , W. Tang , Y. Hu , Y. Zhang , J. Sun , X. Guo , H. Lu , Y. Yang , C. Fang , X. Niu , J. Yue , Z. Fei , Y. Liu , Plant J. 2019, 99, 359. 30912865 10.1111/tpj.14330

[62] Y. Peng , K. Lin‐Wang , J. M. Cooney , T. Wang , R. V. Espley , A. C. Allan , Hortic Res 2019, 6, 3. 30622721 10.1038/s41438-018-0076-4 PMC6312553

[63] S. Nardozza , H. L. Boldingh , M. P. Kashuba , R. Feil , D. Jones , A. H. Thrimawithana , H. S. Ireland , M. Philippe , M. W. Wohlers , T. K. McGhie , M. Montefiori , J. E. Lunn , A. C. Allan , A. C. Richardson , Plant Cell Environ 2020, 43, 819. 31834629 10.1111/pce.13699

[64] J. A. Rodrigues , R. V. Espley , A. C. Allan , Hortic Res 2021, 8, 77. 33790254 10.1038/s41438-021-00514-1 PMC8012628

[65] W.‐Q. Wang , S. M. A. Moss , L. Zeng , R. V. Espley , T. Wang , K. Lin‐Wang , B.‐L. Fu , K. E. Schwinn , A. C. Allan , X.‐R. Yin , New Phytol 2022, 235, 630. 35348217 10.1111/nph.18122

[66] C. Ampomah‐Dwamena , A. H. Thrimawithana , S. Dejnoprat , D. Lewis , R. V. Espley , A. C. Allan , New Phytol 2019, 221, 309. 30067292 10.1111/nph.15362 PMC6585760

[67] M. Tu , Y. Wu , J. Li , D. Chen , G. Jiang , H. Song , X. Yin , X. Liu , M. Li , S. Sun , (49, pg 106,. New Zealand Journal of Crop and Horticultural Science 2020) 2022, 50, 92.

[68] J. Luo , M. Abid , Y. Zhang , X. Cai , J. Tu , P. Gao , Z. Wang , H. Huang , Int. J. Mol. Sci. 2023, 24, 1993. 36768313 10.3390/ijms24031993 PMC9917040

[69] D. Medini , C. Donati , H. Tettelin , V. Masignani , R. Rappuoli , Current Opinion in Genetics & Development 2005, 15, 589. 16185861 10.1016/j.gde.2005.09.006

[70] X. Li , Y Wei , A Acharya , Q Jiang , J Kang , E. A Brummer , G3 Genes|Genomes|Genetics 2014, 4, 1971. 25147192 10.1534/g3.114.012245 PMC4199703

