# Supplementary material for: Graph‐Based Pangenome of Actinidia chinensis Reveals Structural Variations Mediating Fruit Degreening
Source: Adv Sci (Weinh). 2024 May 17;11(28):2400322. doi: 10.1002/advs.202400322 (PMC11267314; doi:10.1002/advs.202400322)
Supplement: Supplementary file 1 — Supporting Information [file ADVS-11-2400322-s001.pdf]

ADVANCED SCIENCE
Open Access

Supporting Information

for *Adv. Sci.*, DOI 10.1002/advs.202400322

Graph-Based Pangenome of *Actinidia chinensis* Reveals Structural Variations Mediating Fruit Degreening

Yingzhen Wang, Pengwei Li, Yanyan Zhu, Feng Zhang, Sijia Zhang, Yan He, Ying Wu, Yunzhi Lin, Hongtao Wang, Wangmei Ren, Lihuan Wang, Ying Yang, Runze Wang, Pengpeng Zheng, Yongsheng Liu, Songhu Wang* and Junyang Yue**

Graph-based pangenome of *Actinidia chinensis* reveals structural variations mediating fruit degreening

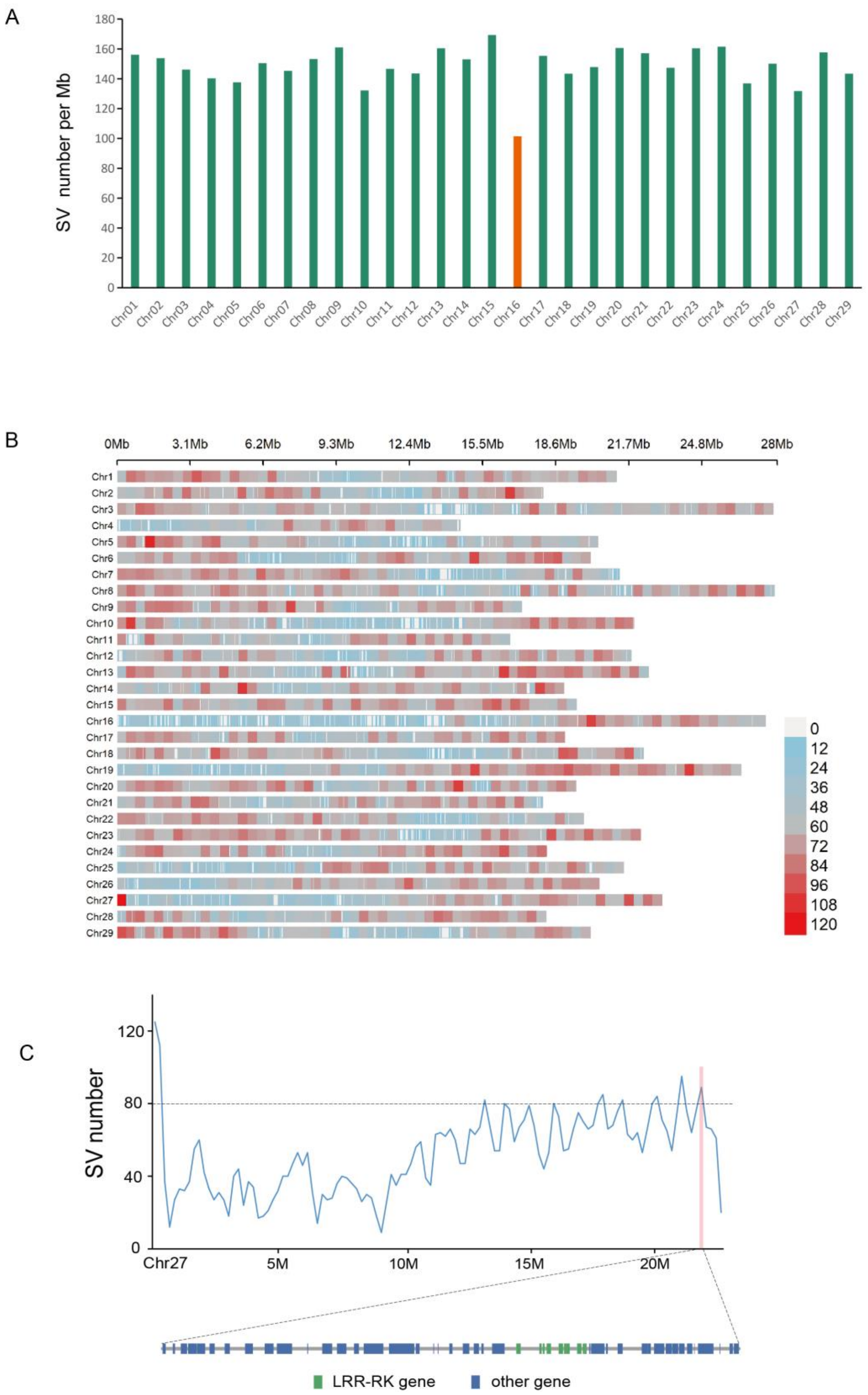

Figure S1 The SV density in kiwifruit genome. (a) The number of SVs per Mb on 29 chromosomes of kiwifruit. (b) The SV density on 29 chromosomes of kiwifruit, the windows size was 400Kb. (c) A representative SV hotspot region on chromosome 27.

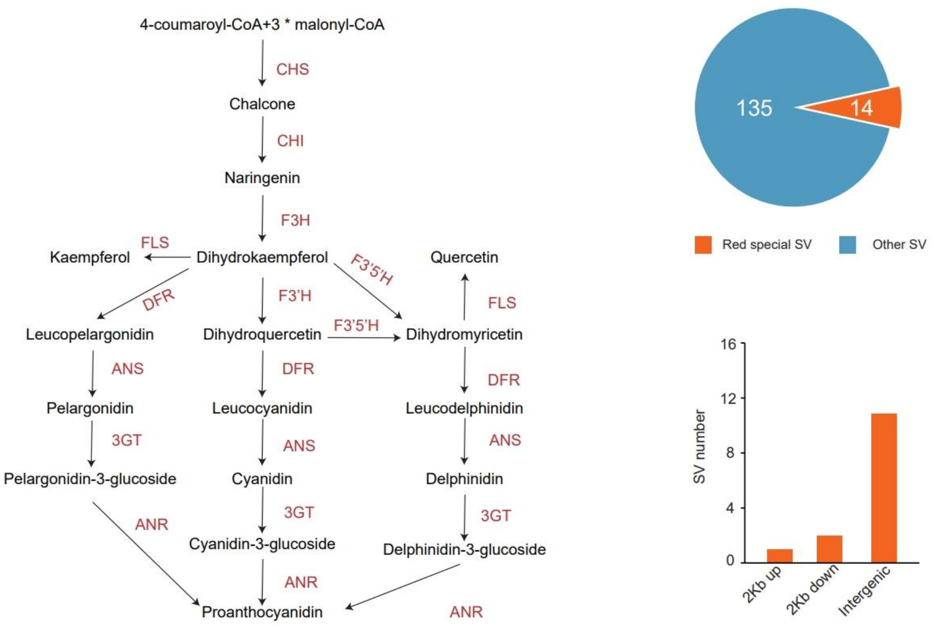

Figure S2 The SVs releated to Anthocyanin synthesis pathway. The piechat represent the number of red fruit group special SVs, the barplots indicated the special SVs sharing overlap with different genomic regions in kiwifruit genome.

Table S1. Summary of PacBio HiFi sequencing data.

Accession	Total bases	Coverage[a] (×)	No. of reads	N50 read length (bp)	Average read length (bp)
Biyu	34,035,495,580	54.9	1,811,467	19,278	18,789
Hort16A	30,250,145,561	48.8	2,024,353	14,866	14,943
Huangyang	25,376,234,346	40.9	1,796,426	14,243	14,126
Jinmi	32,899,320,572	53.1	2,082,440	16,314	15,798
Jinpai	25,334,982,711	40.9	1,439,070	17,370	17,605
Zps18	26,536,276,306	42.8	1,784,143	14,772	14,873

[a]The estimated size of these genomes is all set as 620 Mb.

Table S2. BUSCO assessments for genomic data of the 14 kiwifruit genome assemblies.

Assembly	Complete (C)		Single-copy (S)		Duplicated (D)		Fragmented (F)		Missing (M)	
	Count	%	Count	%	Count	%	Count	%	Count	%
Biyu.hap1	1,604	99.4	1,151	71.3	453	28.1	3	0.2	7	0.4
Biyu.hap2	1,595	98.8	1,146	71.0	449	27.8	6	0.4	13	0.8
Hongyang.hap1	1,602	99.3	1,142	70.8	460	28.5	4	0.2	8	0.5
Hongyang.hap2	1,602	99.3	1,135	70.3	467	29.0	4	0.2	8	0.5
Hort16A.hap1	1,604	99.4	1,135	70.3	469	29.1	3	0.2	7	0.4
Hort16A.hap2	1,603	99.3	1,147	71.1	456	28.3	3	0.2	8	0.5
Huangyang.hap1	1,600	99.1	1,135	70.3	465	28.8	4	0.3	10	0.6
Huangyang.hap2	1,603	99.3	1,137	70.5	466	28.9	3	0.2	8	0.5
Jinmi.hap1	1,606	99.5	1,135	70.3	471	29.2	3	0.2	5	0.3
Jinmi.hap2	1,598	99.0	1,140	70.6	458	28.4	6	0.4	10	0.6
Jinpai.hap1	1,604	99.4	1,137	70.5	467	28.9	3	0.2	7	0.4
Jinpai.hap2	1,600	99.1	1,158	71.8	442	27.4	7	0.4	7	0.4
Zps18.hap1	1,603	99.3	1,135	70.3	468	29.0	6	0.4	5	0.3
Zps18.hap2	1,604	99.4	1,146	71.0	458	28.4	3	0.2	7	0.4

Table S3. Genome-wide identification of SSRs from the 14 kiwifruit genome assemblies.

Assembly	Mono-nucleotide	Di-nucleotide	Tri-nucleotide	Tetra-nucleotide	Penta-nucleotide	Hexa-nucleotide	Compound[a]	Total
Biyu.hap1	172,232	119,820	18,186	5,869	1,810	2,418	8,845	329,180
Biyu.hap2	171,640	119,145	18,022	5,798	1,751	2,327	8,798	327,481
Hongyang.hap1	174,588	121,036	18,236	5,784	1,796	2,263	8,820	332,523
Hongyang.hap2	172,140	119,736	18,080	5,735	1,799	2,226	8,729	328,445
Hort16A.hap1	173,264	120,177	18,048	5,832	1,721	2,196	8,775	330,013
Hort16A.hap2	172,833	119,918	18,097	5,848	1,766	2,188	8,838	329,488
Huangyang.hap1	174,479	120,441	18,202	5,764	1,820	2,190	8,827	331,723
Huangyang.hap2	173,521	120,103	18,116	5,785	1,754	2,152	8,736	330,167
Jinmi.hap1	174,672	121,137	18,161	5,902	1,771	2,234	8,615	332,492
Jinmi.hap2	171,294	119,478	18,107	5,675	1,712	2,204	8,699	327,169
Jinpai.hap1	173,534	120,418	18,439	5,910	1,829	2,393	8,924	331,447
Jinpai.hap2	170,651	119,330	18,051	5,791	1,740	2,364	8,801	326,728
Zps18.hap1	174,435	120,618	18,182	5,798	1,819	2,136	8,812	331,800

Zps18.hap2	173,986	120,906	18,114	5,898	1,789	2,202	8,865	331,760

[a]Compound SSRs were defined as those with an interval less than 100 nt between the two continuous repeat motifs.

Table S4. Statistics of predicted protein-coding genes in the 14 kiwifruit genome assemblies.

Assembly	No. of predicted genes	Average gene length (bp)	No. of predicted transcripts	Average transcript length (bp)	Average CDS length (bp)	No. of exons per transcript	Average intergenic space distance (bp)	Average intron length (bp)
Biyu.hap1	46,073	5,319	51,425	5,592	1,173	5.9	7,735	911
Biyu.hap2	46,634	5,314	52,127	5,609	1,176	5.9	7,493	911
Hongyang.hap1	45,809	5,284	51,252	5,539	1,164	5.8	7,925	913
Hongyang.hap2	45,434	5,405	51,215	5,703	1,176	6.0	7,777	908
Hort16A.hap1	44,336	5,522	49,633	5,813	1,199	6.0	8,059	920
Hort16A.hap2	46,230	5,306	51,327	5,554	1,167	5.8	7,686	922
Huangyang.hap1	45,982	5,348	51,759	5,644	1,173	5.9	7,787	911
Huangyang.hap2	45,004	5,467	50,623	5,761	1,192	6.0	7,870	915
Jinmi.hap1	45,473	5,418	51,026	5,704	1,182	5.9	7,851	919
Jinmi.hap2	45,101	5,374	50,317	5,656	1,182	5.9	7,818	918
Jinpai.hap1	46,244	5,309	51,306	5,559	1,169	5.8	7,728	923
Jinpai.hap2	45,675	5,234	50,861	5,490	1,161	5.8	7,732	909

Zps18.hap1	45,873	5,315	51,185	5,570	1,171	5.8	7,798	918
Zps18.hap2	45,481	5,352	50,674	5,614	1,176	5.8	7,882	921

Table S5. BUSCO assessments for predicted genes of the 14 kiwifruit genome assemblies.

Assembly	Complete (C)		Single-copy (S)		Duplicated (D)		Fragmented (F)		Missing (M)	
	Count	%	Count	%	Count	%	Count	%	Count	%
Biyu.hap1	1,558	96.5	1,011	62.6	547	33.9	37	2.3	19	1.2
Biyu.hap2	1,552	96.2	1,006	62.3	546	33.8	34	2.1	28	1.7
Hongyang.hap1	1,564	96.9	1,032	63.9	532	33.0	28	1.7	22	1.4
Hongyang.hap2	1,562	96.8	1000	62.0	562	34.8	29	1.8	23	1.4
Hort16A.hap1	1,568	97.2	1,015	62.9	553	34.3	28	1.7	18	1.1
Hort16A.hap2	1,561	96.7	1,022	63.3	539	33.4	29	1.8	24	1.5
Huangyang.hap1	1,558	96.5	988	61.2	570	35.3	33	2.0	23	1.4
Huangyang.hap2	1,563	96.8	1,008	62.5	555	34.4	30	1.9	21	1.3
Jinmi.hap1	1,561	96.7	999	61.9	562	34.8	29	1.8	24	1.5
Jinmi.hap2	1,545	95.7	988	61.2	557	34.5	35	2.2	34	2.1
Jinpai.hap1	1,560	96.7	1,004	62.2	556	34.5	35	2.2	19	1.2
Jinpai.hap2	1,556	96.4	1,022	63.3	534	33.1	35	2.2	23	1.4
Zps18.hap1	1,545	95.7	1,001	62.0	544	33.7	43	2.7	26	1.6
Zps18.hap2	1,558	96.5	993	61.5	565	35.0	25	1.6	31	1.9

Table S6. Non-redundant kiwifruit pan-genome based on protein-coding genes.

Assembly	No. of total genes	No. of core genes	No. of softcore genes	No. of shell genes	No. of cloud genes[a]	Core/pan ratio (%)
Biyu.hap1	46,073	29,350	6,813	9,421	489	63.7
Biyu.hap2	46,634	29,407	6,669	9,927	631	63.1
Hongyang.hap1	45,809	29,465	6,871	8,919	554	64.3
Hongyang.hap2	45,434	29,302	6,748	8,850	534	64.5
Hort16A.hap1	44,336	29,184	6,491	7,930	731	65.8
Hort16A.hap2	46,230	29,482	6,673	9,177	898	63.8
Huangyang.hap1	45,982	29,367	6,807	9,198	610	63.9
Huangyang.hap2	45,004	29,271	6,692	8,394	647	65.0
Jinmi.hap1	45,473	29,284	6,790	8,740	659	64.4
Jinmi.hap2	45,101	29,301	6,608	8,514	678	65.0
Jinpai.hap1	46,244	29,576	6,922	9,288	458	64.0
Jinpai.hap2	45,675	29,418	6,663	9,108	486	64.4
Zps18.hap1	45,873	29,447	6,677	8,889	860	64.2
Zps18.hap2	45,481	29,406	6,795	8,587	693	64.7

Pan-genome[b]	49,770	23,217	6,278	20,249	26	46.6

[a]The unassigned genes in each assembly were grouped into one cluster in our study.

[b]The representative genes in each cluster of our constructed pan-genome were counted.

Table S7. Statistics of gene length, expression level, and K_a/K_s of representive genes in different cluster categories.

Category	Core	Dispensable	Softcore	Shell	Cloud
Number of pan-genes	23,217	26,553	6,278	20,249	26
Average length of cDNA sequences	6,379	3,600	4,593	3,282	4,528
Average length of CDS sequences	1,411	707	1,035	602	892
Average count in gene expression	1,052	326	640	226	256
Number of gene pairs used to compute K_a/K_s	307,507	162,532	84,461	74,291	3,780
Average value of K_a/K_s	0.434	0.510	0.480	0.555	0.350

Table S8. The top 10 enriched GO terms of biological process and molecular function categories for the core genes.

GO term	GO description	Count	Proportion (%)	*P*-value
Biological process:				
GO:0045595	Regulation of cell differentiation	65	78.3	0.00013857
GO:0030435	Sporulation resulting in formation of a cellular spore	104	73.8	0.00014643
GO:0009938	Negative regulation of gibberellic acid mediated signaling pathway	132	71.7	0.00017549
GO:0007219	Notch signaling pathway	24	92.3	0.00017762
GO:0045893	Positive regulation of transcription, DNA-templated	1,003	63	0.00025792
GO:0050896	Response to stimulus	62	77.5	0.00033363
GO:0043507	Positive regulation of JUN kinase activity	43	81.1	0.00047099
GO:2000022	Regulation of jasmonic acid mediated signaling pathway	159	69.4	0.00054744
GO:2000146	Negative regulation of cell motility	32	84.2	0.00073258
GO:0001568	Blood vessel development	34	82.9	0.00087803
Molecular function:				
GO:0000976	Transcription regulatory region sequence-specific DNA binding	1,237	64.4	0
GO:0003700	Transcription factor activity, sequence-specific DNA binding	1,438	62.6	0

GO:0043565	Sequence-specific DNA binding	916	64.8	0
GO:0033612	Receptor serine/threonine kinase binding	100	69.9	0.00026359
GO:0004713	Protein tyrosine kinase activity	188	65.5	0.00031589
GO:0017046	Peptide hormone binding	37	80.4	0.00034725
GO:0051020	GTPase binding	43	76.8	0.00075416
GO:0003682	Chromatin binding	390	61.7	0.00076552
GO:0000978	RNA polymerase II core promoter proximal region sequence-specific DNA binding	281	62.9	0.0008238
GO:0004706	JUN kinase kinase kinase activity	34	79.1	0.00107832

Table S9. The top 10 enriched GO terms of biological process and molecular function categories for the dispensable genes.

GO term	GO description	Count	Proportion (%)	*P*-value
Biological process:				
GO:0005985	Sucrose metabolic process	236	57.3	0
GO:0006508	Proteolysis	515	55.3	0
GO:0006886	Intracellular protein transport	294	54.3	0
GO:0015074	DNA integration	412	89.8	0
GO:0055085	Transmembrane transport	357	54.1	0
GO:0055114	Oxidation-reduction process	957	53.9	0
GO:0019253	Reductive pentose-phosphate cycle	35	68.6	0.000101
GO:0071669	Plant-type cell wall organization or biogenesis	51	63	0.000102
GO:0015031	Protein transport	414	48.1	0.000108
GO:0060627	Regulation of vesicle-mediated transport	37	67.3	0.000124
Molecular function:				
GO:0003676	Nucleic acid binding	807	67.4	0
GO:0003964	RNA-directed DNA polymerase activity	164	86.3	0
GO:0004190	Aspartic-type endopeptidase activity	210	73.9	0

GO:0004523	RNA-DNA hybrid ribonuclease activity	76	80.9	0
GO:0005524	ATP binding	2,346	51.6	0
GO:0080043	Quercetin 3-O-glucosyltransferase activity	101	59.8	0.000105
GO:0080044	Quercetin 7-O-glucosyltransferase activity	96	60	0.000125
GO:0033897	Ribonuclease T2 activity	16	88.9	0.000156
GO:0016887	ATPase activity	472	51.1	0.000208
GO:0047213	Anthocyanidin 3-O-glucosyltransferase activity	30	73.2	0.000272

Table S10. The top 10 enriched KEGG pathways for the core genes.

KEGG ID	KEGG description	Count	Proportion (%)	*P*-value
map00511	Other glycan degradation	19	0.54503730	0.00318686
map00520	Amino sugar and nucleotide sugar metabolism	78	2.23752152	0.03319580
map00908	Zeatin biosynthesis	24	0.68846816	0.03390191
map00563	Glycosylphosphatidylinositol (GPI)-anchor biosynthesis	22	0.63109581	0.03748915
map04210	Apoptosis	43	1.23350545	0.03763528
map04016	MAPK signaling pathway - plant	106	3.04073437	0.04509223
map00040	Pentose and glucuronate interconversions	69	1.97934596	0.05193249
map00100	Steroid biosynthesis	24	0.68846816	0.05598404
map00905	Brassinosteroid biosynthesis	23	0.65978199	0.06913698
map03008	Ribosome biogenesis in eukaryotes	64	1.83591509	0.09138188

Table S11. The top 10 enriched KEGG pathways for the dispensable genes.

KEGG ID	KEGG description	Count	Proportion (%)	*P*-value
map00140	Steroid hormone biosynthesis	34	1.02967898	0.01258801
map00910	Nitrogen metabolism	23	0.69654755	0.01598465
map00943	Isoflavonoid biosynthesis	18	0.54512417	0.01755945
map01212	Fatty acid metabolism	46	1.39309509	0.01872840
map00061	Fatty acid biosynthesis	30	0.90854028	0.02738521
map00220	Arginine biosynthesis	25	0.75711690	0.02913591
map00630	Glyoxylate and dicarboxylate metabolism	51	1.54451847	0.03279202
map00240	Pyrimidine metabolism	82	2.48334343	0.04961891
map04550	Signaling pathways regulating pluripotency of stem cells	15	0.45427014	0.06353985
map01040	Biosynthesis of unsaturated fatty acids	17	0.51483949	0.06489447
